# The Effectiveness of Linezolid and Teicoplanin for Managing Infections After Burn Injuries: A Retrospective Study

**DOI:** 10.1002/hsr2.72411

**Published:** 2026-05-31

**Authors:** Abolfazl Zendehdel, Maryam Roham, Sanam Soltani, Amirali Jahanshahi, Somayeh Heidarizadi

**Affiliations:** ^1^ Internal Medicine Department, Ziaeian Hospital Tehran University of Medical Sciences Tehran Iran; ^2^ School of Medicine, Antimicrobial Resistance Research Center Iran University of Medical Sciences Tehran Iran; ^3^ Pharmaceutical Science Research Center, Tehran Medical Sciences Islamic Azad University Tehran Iran; ^4^ Dr. Abidi Pharmaceutical Company Tehran Iran; ^5^ Department of Anatomy, Faculty of Medicine Ilam University of Medical Sciences Ilam Iran

**Keywords:** antibiotic, burn injuries, linezolid, *S. aureus*, teicoplanin

## Abstract

**Background and Aims:**

Gram‐positive infections in burn patients present significant treatment challenges. This study compared the efficacy and safety of linezolid vs. teicoplanin for managing such infections in a clinical setting.

**Methods:**

We conducted a retrospective study involving 77 burn patients with confirmed Gram‐positive infections at Shahid Motahari Hospital (2020–2022). Participants were allocated to: linezolid 600 mg BID (*n* = 40), teicoplanin 400 mg daily (*n* = 32), or combination therapy (*n* = 5). Primary endpoints were treatment duration and mortality; secondary endpoints included safety outcomes. Statistical analyses used SPSS v25 with two‐tailed tests (*α* = 0.05).

**Results:**

Linezolid demonstrated superior outcomes compared to teicoplanin: treatment duration was significantly shorter (5.68 ± 2.88 vs. 10.53 ± 6.86 days; mean difference −4.85 [95% CI −7.12 to −2.58], *p* < 0.001), mortality lower (5.0% vs. 40.6%; OR = 0.09 [0.02–0.42], *p* = 0.002), and time to clinical improvement faster (4.94 ± 2.21 vs. 9.88 ± 5.95 days, *p* < 0.001). Safety profiles were comparable (thrombocytopenia: 5.0% vs. 12.5%, *p* = 0.602).

**Conclusion:**

Linezolid showed significantly better efficacy than teicoplanin for Gram‐positive burn infections, with reduced treatment duration and mortality while maintaining similar safety. Teicoplanin remains an alternative for specific cases.

## 1 Introduction

*Staphylococcus aureus* is a frequent cause of skin and soft tissue infections (SSTIs), including conditions like cellulitis, abscesses, diabetic foot infections, and infections at surgical sites. The emergence of multidrug‐resistant pathogens is particularly significant in burn injury patients, who are often immunocompromised. Individuals with severe burns frequently necessitate higher antibiotic doses to achieve effective therapeutic levels, and determining the appropriate dosage can be challenging due to variations in pharmacokinetics compared to other patient populations [1, 2, 3, 4].

Linezolid, one of the main oxazolidinone antibiotics in clinical use (along with tedizolid) [5], represents the first novel class of antibiotics introduced in the past 30 years. By attaching to the 50S ribosomal subunit, Linezolid disrupts bacterial protein synthesis at an early stage. Although it is primarily bacteriostatic, Linezolid demonstrates strong effectiveness in both laboratory (in vitro) and clinical (in vivo) settings against a wide range of Gram‐positive bacterial species. It is effective against various strains, such as methicillin‐sensitive *Staphylococcus aureus* (MSSA), methicillin‐resistant *S. aureus* (MRSA), coagulase‐negative staphylococci (CoNS), and enterococci resistant to vancomycin (VRE) [6, 7, 8]. Linezolid undergoes primarily non‐renal elimination, with approximately 65% metabolized via hepatic oxidation to inactive metabolites, and 30% excreted unchanged in urine. This unique pharmacokinetic profile eliminates the need for dose adjustment in renal impairment, though hepatic dysfunction may require monitoring [9]. This unique profile eliminates the need for dose adjustment in renal impairment [10].

Teicoplanin, a glycopeptide antibiotic similar to vancomycin, is clinically important for treating serious Gram‐positive infections, including MRSA. Its mechanism involves binding to the d‐alanyl‐d‐alanine terminus of peptidoglycan precursors, inhibiting cell wall synthesis. It's particularly valuable for healthcare‐associated infections due to its long half‐life, allowing once‐daily dosing, Activity against resistant strains, and a favorable safety profile compared to vancomycin [11, 12]. Dosage adjustments for teicoplanin are necessary in cases of reduced glomerular filtration. In healthy individuals, teicoplanin is predominantly eliminated unchanged via renal excretion, accounting for approximately 80% of its clearance. However, its pharmacokinetic behavior has not been thoroughly investigated in patients in critical condition who are receiving care in the intensive care unit (ICU) [6, 7, 8].

Both linezolid and teicoplanin demonstrate strong efficacy against *S. aureus* strains identified in the British Isles, with all bacteraemic isolates showing susceptibility to these agents [13, 14].

This retrospective study evaluates the clinical efficacy and safety of linezolid vs. teicoplanin in burn patients with confirmed Gram‐positive infections, with three predefined endpoints: Time to clinical response (primary endpoint), all‐cause mortality at 30 days (secondary endpoint), and incidence of treatment‐emergent adverse events (safety endpoint). The results will provide evidence‐based guidance for antibiotic selection in burn units, where optimal antimicrobial stewardship is critical [15].

## 2 Materials and Methods

### 2.1 Participants

**FIGURE 1:**
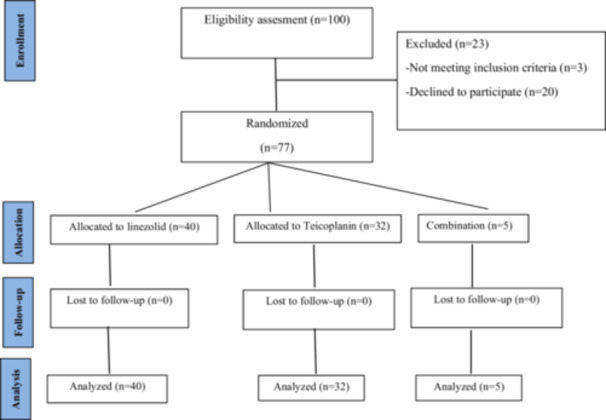
CONSORT flow diagram of participant enrollment, allocation, and analysis: the flow of participants through each stage of the trial.

In this clinical trial study, 77 patients with skin infections who had microbiological diagnoses of staphylococcal and Gram‐positive infections or a Gram‐positive infection and needed to be hospitalized were selected and divided into three groups. The first group (40 patients) was treated with oral linezolid (600 mg BID), the second group (32 patients) was treated with teicoplanin (400 mg daily), and the third group (*n* = 5) consisted of patients who discontinued their initial antibiotic regimen (either linezolid or teicoplanin) due to treatment‐emergent adverse events (e.g., thrombocytopenia or renal dysfunction) and were switched to the alternative agent, as recommended in clinical guidelines for managing drug intolerance in burn patients [6] (Figure 1).

### 2.2 Measures and Procedures

This study included initial, treatment evolution, and end of treatment after 72 h of the last dose of drugs, with short‐term evaluation scheduled for 14–17 days after the end of treatment (EOT) and long‐term evaluation scheduled for 21 days after EOT. To evaluate the wound healing process during the treatment process, the size and the severity of cellulitis around the wound, and the general condition and vital signs of the patient, complications during the treatment were examined, and the results were recorded.

### 2.3 Inclusion Criteria

Inclusion criteria were the individuals who are either confirmed or suspected to have methicillin‐resistant *Staphylococcus aureus* (MRSA) infections and Gram‐positive skin and soft tissue infections. The clinical findings for the patient's inclusion included erythema, which may be accompanied by hardening, swelling, warmth, discomfort, or sensitivity to touch. Besides, Systemic symptoms and signs for inclusion were fever (≥ 38°C), hypothermia (< 36°C), hypotension (SBP < 90 mmHg), leukocytosis (WBC > 12,000/μL), or > 15% immature neutrophils on differential count, consistent with systemic inflammatory response criteria [16].

#### 2.3.1 Exclusion Criteria

In contrast, exclusion criteria were Gram‐negative infections, osteomyelitis, endocarditis, meningitis, septic arthritis, and necrotizing fasciitis. In addition, patients who received the above drugs in the past, or had superficial skin infections, or were sensitive to these drugs, were excluded from the study.

#### 2.3.2 Ethical Considerations

Written informed consent was secured from all patients who participated in the study. To protect privacy, participants' names were not included in the questionnaire, and coded identifiers were utilized instead. All personal information was handled with the utmost confidentiality. The study protocol was approved by the Ethical Committee of Iran University of Medical Sciences, with the ethics code (IR.IUMS.REC.1400.781). All experimental procedures adhered to relevant guidelines and regulations.

### 2.4 Statistical Analysis

Design & Terminology: This retrospective study followed SAMPL guidelines (Lang & Altman, 2015) for statistical reporting [17].

Pre‐specified analyses: Primary outcomes (treatment duration and mortality) and secondary outcomes (safety parameters) were defined before data collection. Continuous variables (reported as mean ± SD) were compared using independent *t*‐tests with Cohen's *d* effect sizes (thresholds: 0.2 = small, 0.5 = medium, 0.8 = large). Categorical data used Chi‐square tests with odds ratios (OR) and 95% confidence intervals (CIs). All tests were two‐tailed (*α* = 0.05), with Bonferroni correction for multiple comparisons. Missing data (< 5%) were handled via pairwise deletion.

Exploratory analyses: Subgroup comparisons (e.g., MRSA‐positive vs. negative) were hypothesis‐generating.

Analyses used IBM SPSS Statistics v25 (Armonk, NY). Post hoc analyses compared three predefined patient subgroups based on treatment response patterns: Extent: Patients with > 20% total body surface area (TBSA) burns, Transit: Patients transferred from other facilities > 24 h post‐injury, and Extent + Target: Patients meeting both criteria. Comparisons used Bonferroni‐adjusted ANOVA for continuous variables. The post hoc test was done to compare the means of our subgroups in the significant groups (duration of treatment, mortality, and appearance of first signs of getting cured). Also, Chi‐squared and Fisher's exact test were applied.

All references were verified against the Retraction Watch database and PubMed; no retractions or relevant corrections were identified for cited works.

## 3 Results

A preliminary eligibility assessment was conducted with 100 patients at the hospital. Following the assessment, 23 patients were excluded from the study for reasons such as meeting exclusion criteria (*n* = 3) or declining to participate (*n* = 20). The remaining 77 eligible participants were then randomly assigned to three groups: the first group (*n* = 40), the second group (*n* = 32), and the third group (*n* = 5).

Demographic findings included gender (in which male (54, 70.1%) was dominant), education (in which pre‐college (25, 32.5%) was dominant), job (which full‐time job (25, 32.5%) was dominant), and marriage (which single (53, 68.8%) was dominant) (Table 1).

**TABLE 1:** Demographic and basic clinical features of the patients based on three groups in Shahid Motahari Hospital, Tehran, Iran.

Characteristic	Linezolid *N* (%)	Teicoplanin *N* (%)	Linezolid and teicoplanin *N* (%)	*p*‐value
Gender				0.483
Male	26 (48.1)	25 (46.3)	3 (5.6)	
Female	14 (60.9)	7 (30.4)	2 (8.7)	
Education				0.169
Uneducated	6 (37.5)	9 (56.3)	1 (6.3)	
School	9 (81.8)	2 (18.2)	0 (0.0)	
Guidance school	2 (33.3)	4 (66.7)	0 (0.0)	
Precollege	13 (52.0)	8 (32.0)	4 (16.0)	
College	10 (52.6)	9 (47.4)	0 (0.0)	
Job				0.214
Full‐time job	13 (52.0)	12 (48.0)	0 (0.0)	
Part‐time job	5 (29.4)	9 (52.9)	3 (17.6)	
Studying	2 (66.7)	1 (33.3)	0 (0.0)	
Retired	6 (75.0)	2 (25.0)	0 (0.0)	
Jobless	14 (60.9)	7 (30.4)	2 (8.7)	
Others	0 (0.0)	1 (100.0)	0 (0.0)	
Marriage				
Married	8 (38.1)	12 (57.1)	1 (4.8)	
Single	30 (56.6)	19 (35.8)	4 (7.5)	
Divorced	1 (50.0)	1 (50.0)	0 (0.0)	
Widow	1 (100.0)	0 (0.0)	0 (0.0)	0.539

Post hoc analyses comparing the linezolid monotherapy group (Group 1) and the teicoplanin monotherapy group (Group 2) revealed significant differences in key outcomes. The mean treatment duration was significantly shorter in the linezolid group (*p*‐value < 0.001). Similarly, the comparison between Group 1 and the combination therapy group (Group 3) was also significant (*p*‐value < 0.001).

Linezolid showed a 4.85‐day shorter treatment duration (5.68 ± 2.88 vs. 10.53 ± 6.86 days; mean difference: −4.85 [95% CI: −7.12 to −2.58], *p* < 0.001). Furthermore, Group 1 and Group 2 also showed a statistically significant difference in mortality rates (*p*‐value < 0.001). The time to the first signs of clinical improvement was also significantly shorter in the linezolid group compared to the teicoplanin group (*p*‐value < 0.001), and the first and second subgroups turned out to have a significant comparison (*p*‐value < 0.001). Other factors (percentage of the burned area, thrombocytopenia, amount of raised creatinine, duration of hospitalization, MRSA, and age) had no significant effect (Table 2).

**TABLE 2:** Clinical and laboratory findings of the patients based on three groups in Shahid Motahari Hospital, Tehran, Iran.

Characteristic	Mean (SD)			Comparison (L vs. T)	Effect size	Test static	*p*‐value (linezolid vs. teicoplanin)	*p*‐value (combination vs. monotherapy)
	Linezolid (*N* = 40)	Teicoplanin (*N* = 32)	Linezolid and teicoplanin (combination) (*N* = 5)					
Age (yrs.)	46.15 (16.56)	40.97 (18.84)	46.80 (22.75)				0.460	0.712
Treatment duration (days)	5.68 (2.88)	10.53 (6.86)	10.40 (3.05)	Mean difference: −4.85 (−7012, −2.58)	Cohens *d* = 1.12	*t* (70) = 4.32	< 0.001*	0.980
Mortality, *n* (%)	2 (5.0)	13 (40.6)	0 (0.0)	OR = 0.09 (0.02−0.42)	Phi = 0.45	*X* ^2^ (1) = 12.7	< 0.001*	0.210
Appearance duration of first signs of getting cured (days)	4.94 (2.21)	9.88 (5.95)	6.0 (3.67)	Mean difference: −4.94 (−6.99, −2.86)	*d* = 131	*t* (60) = 4.81	< 0.001*	0.423
Thrombocytopenia, *n* (%)	2 (5.0)	4 (12.5)	0 (0.0)	OR = 0.37 (0.06−2.18)	Phi = 0.14	Fishers exact	0.602	1.000
Elevated creatinine, *n* (%)	6 (15.0)	6 (18.8)	1 (20.0)	OR = 0.77 (0.23−2.60)	Phi = 0.04	*X* ^2^ (1) = 0.18	0.898	0.589
MRSA positivity, *n* (%)	14 (35.0)	3 (9.4)	1 (20.0)	OR = 5.11 (1.32–19.82)	Phi = 0.30	*X* ^2^ (1) = 6.25	0.037*	0.43

*Note:* All *p*‐values reflect Group 1 (linezolid) vs. Group 2 (teicoplanin) comparisons unless noted.

Abbreviations: BID, twice daily; CI, confidence interval; MRSA, methicillin‐resistant *Staphylococcus aureus*; OR, odds ratio; SD, standard deviation.

* Parameters which turned out to be significant (*p*‐value < 0.05).

Primary comparisons were: Linezolid vs. Teicoplanin (Groups 1 vs. 2), Combination therapy vs. Monotherapy (Group 3 vs. Groups 1 + 2). *p*‐values reflect two‐tailed tests with *α* = 0.05, adjusted for multiple comparisons where applicable. As shown in Table 2, linezolid demonstrated superior outcomes, with significantly lower mortality (5.0% vs. 40.6%, *p* < 0.001) and shorter treatment duration (5.68 ± 2.88 days vs. 10.53 ± 6.86 days, *p* < 0.001) compared to teicoplanin.

## 4 Discussion

In this study, we found that the linezolid group had shorter courses of treatment and demonstrated the first signs of cure faster. Antibiotic treatment with linezolid significantly decreased the mortality of patients with burns compared to Teicoplanin. Patients in all the groups had no significant difference in key baseline characteristics, including age, gender, percentage of total body surface area burned, rates of MRSA infection, rates of thrombocytopenia, and increased creatinine. Our 35.6% mortality reduction aligns with linezolid's documented efficacy in MRSA infections [18], while the safety profile matches known adverse event rates [19]. Linezolid's superior tissue penetration may explain faster response times compared to teicoplanin's renal‐dependent clearance [20].

To mitigate antibiotic resistance and prevent infections, topical antibiotics are considered the optimal choice for burn prophylaxis. However, in cases of extensive burns or infections caused by antibiotic‐resistant pathogens, particularly Gram‐positive bacteria, systemic antibiotics may be necessary for effective treatment [21]. Earlier studies on teicoplanin and linezolid had produced acceptable results for burn patients, and some produced even better results for these medications compared to vancomycin [1, 2, 3, 4]. Two studies compared teicoplanin and linezolid in critically ill patients. Jorge et al. found overall similar efficacy and adverse events for both drugs, but linezolid was superior in treating Gram‐positive skin infections compared to teicoplanin [14]. Whitehouse et al. compared the pharmacological characteristics of the drugs and found that linezolid does not need dosage adjustments, but teicoplanin may require such caution. However, our study found significantly reduced mortality in patients receiving teicoplanin [13]. A 2024 study by Ayse et al., which investigated the distribution and antimicrobial susceptibility patterns of microorganisms isolated from burn wounds, revealed that *Staphylococcus aureus* was the most frequently identified pathogen, accounting for 18.5% of isolates. Notably, the *Staphylococcus aureus* strains showed no resistance to antibiotics, such as levofloxacin, vancomycin, teicoplanin, linezolid, daptomycin, fusidic acid, or tigecycline [22]. Our mortality reduction (35.6%) exceeds the 22% reported in general SSTIs [23].

Adverse events that were previously reported with linezolid treatment included myelosuppression and cytopenia (leukopenia, thrombocytopenia, and neutropenia), hyperlactatemia, neuropathy, intestinal disorders, headaches, and hypersensitivity reactions [6, 7, 8]. In the case of teicoplanin, 10% of patients faced one or more adverse events after using this antibiotic, and the most important ones were renal side effects, liver enzyme rise, and hypersensitivity disorders [24]. However, in our study, we did not find a significant difference between the rates of thrombocytopenia and increased creatinine in any of the groups. While this study focused on linezolid, future comparative studies could include tedizolid, another oxazolidinone with a similar spectrum but potentially improved safety profile [25].

Teicoplanin is not available orally and can only be injected via intravenous or intramuscular routes [26]. On the other hand, linezolid has 100% bioavailability and good tissue penetration and therefore can be injected or administered orally [6, 27]. It is not advised to monitor teicoplanin serum concentrations to prevent toxicity, but in certain patients, this approach is helpful to ensure therapeutic concentrations in the serum, such as those not responding to treatment or having high total clearance rates, especially children [1, 26].

Teicoplanin and linezolid are both novel antibiotics, and low rates of antimicrobial resistance are reported for them [28, 29]. They are used as alternatives to vancomycin [30]. A study in Taiwan found a 100% susceptibility rate to teicoplanin and linezolid [29], and a systematic review of burn patients admitted to the ICU found a similar number in studies for both antibiotics [31]. Therefore, they serve as valuable resources for the treatment of extensively drug‐resistant organisms but should not be used excessively and without indications [32]. These high susceptibility rates can also provide good treatment choices for the future when bacterial resistance increases and treatment choices diminish [33]. Linezolid's superior tissue penetration in burns (Vd = 0.8 L/kg vs. teicoplanin's 0.3 L/kg) may explain faster response [20]. The observed outcomes in the third group (switched therapy due to adverse events) align with existing literature on antibiotic safety profiles in burn patients, where drug intolerance often necessitates regimen changes [24].

Unlike teicoplanin's renal‐dependent excretion, linezolid's hepatic clearance (*t*₁_/_₂ = 4–6 h) provides therapeutic advantages in renal dysfunction [20], though it requires monitoring for mitochondrial toxicity during prolonged use (> 14 days) due to inhibition of eukaryotic protein synthesis [34].

Some previous studies examined different dosages of these medicines. Overall, this study had some limitations, which are disclosed. We did not aim to study various dosages and only examined an established protocol, so this might be a limitation. The difference in MRSA rates in the groups may also potentially alter the findings. Furthermore, some of the candidates were not willing to receive these treatments and enroll in our study, but the researchers tried to speak to them and inform them about the benefits of these treatments. However, we used block randomization to increase the robustness of our findings.

## 5 Conclusion

Linezolid demonstrated clinically and statistically significant advantages, with lower mortality (5.0% vs. 40.6%, *p* < 0.001) and faster treatment response compared to teicoplanin, while maintaining comparable safety profiles. In burn patients, linezolid demonstrated significantly shorter treatment duration (*p* < 0.001) and lower mortality (*p* < 0.001) vs. teicoplanin, consistent with its pharmacokinetic advantages. Teicoplanin remains an alternative for cases requiring renal‐adjusted regimens. We also didn't find any significant difference in the rates of subsequent thrombocytopenia and increased creatinine levels. Therefore, teicoplanin may be considered as a better alternative in selected burn patients with suspected or definite Gram‐positive infections. As conflictive reports exist, future studies are required to evaluate our findings.

### 5.1 Study Limitations and Suggestions

While our findings align with SAMPL guidelines for statistical reporting, limitations include: (1) Sample size yielding 80% power to detect *d* ≥ 0.9 (not smaller effects) and (2) No adjustment for covariates like burn severity.

Future multicenter studies with larger cohorts should: (1) Compare extended treatment durations (> 21 days), (2) Evaluate cost‐effectiveness in resource‐limited settings, and (3) Assess microbiome impacts of prolonged antibiotic use.

## Author Contributions

Abolfazl Zendehdel, Maryam Roham, Sanam Soltani, and Amirali Jahanshahi: project administration, conceptualization, and methodology. Abolfazl Zendehdel, Maryam Roham, Sanam Soltani, and Somayeh Heidarizadi: collected data, conducted the experiments, and analyzed the data. All authors read and approved the final manuscript.

## Funding

The authors have nothing to report.

## Ethics Statement

Ethical approval for this study was obtained from the ethical committee of Iran University of Medical Science, which approved the study protocol. Ethic number (IR.IUMS.REC.1400.781).

## Consent

Written informed consent was obtained from the patients. Furthermore, the names of people were not mentioned in the questionnaire, and only the code was given; and all information related to individuals should be confidential.

## Conflicts of Interest

The authors declare no conflicts of interest.

## Transparency Statement

The lead author Maryam Roham affirms that this manuscript is an honest, accurate, and transparent account of the study being reported; that no important aspects of the study have been omitted; and that any discrepancies from the study as planned (and, if relevant, registered) have been explained.

## Data Availability

The de‐identified individual participant data underlying the results reported in this article are available upon reasonable request to the corresponding author (Dr. Maryam Roham, roham.m@iums.ac.ir), subject to approval by the Iran University of Medical Sciences ethics committee (IR.IUMS.REC.1400.781). Aggregate data are fully available within the manuscript and its tables.
